# Systematically Prognostic Analyses of Gastric Cancer Patients with Ovarian Metastasis

**DOI:** 10.1155/2023/9923428

**Published:** 2023-04-30

**Authors:** Peng Peng, Xiuyuan Liu, Lin Yang, Zhenguang Gu, Lin Cai

**Affiliations:** ^1^Department of General Surgery, Xuzhou Kuangshan Hospital, Xuzhou, China; ^2^School of Food and Drug, Xuzhou Polytechnic College of Bioengineering, Xuzhou, China

## Abstract

Ovarian metastasis of gastric cancer indicates that the disease has reached the late stage and the opportunity for radical surgery is restricted. However, the clinical characteristics and prognosis of patients with gastric cancer ovarian metastasis (GCOM) remain to be illustrated. Here, we retrieved the information of 780 GCOM cases from the Surveillance, Epidemiology, and End Results (SEERs) database and analyzed their clinicopathological characteristics as well as their survival. According to our data, most GCOM patients showed poor pathological differentiation, advanced T and N stages. The prognostic factors include patients' age, tumor size, surgical resection, and chemotherapy treatment. Of note, the marriage status was also identified as an independent prognostic factor. Besides the identification of prognostic factors, we established nomograms to help predict the overall survival and cancer-specific survival of GCOM, respectively.

## 1. Introduction

Gastric cancer represents a common malignancy worldwide [1]. Because the clinical symptoms of early gastric cancer are not typical, many patients are already in the middle and late stages when they seek treatment and have lost the opportunity for radical surgery. Currently, the main ways of gastric cancer metastasis include lymphatic metastasis, hematologic metastasis, and peritoneal implantation [2, 3]. Among them, ovarian metastasis of gastric cancer is mostly caused by peritoneal implantation metastasis. After the cancer tissue invades the serosa, it falls off to the peritoneal cavity to cause implantation metastasis [4, 5].

Ovarian metastases from gastric cancer are clinically known as Krukenberg tumor, which can be mucinous cell carcinoma, poorly differentiated adenocarcinoma, or tubular adenocarcinoma [6]. Once gastric cancer has ovarian metastasis, it means that the disease has reached the late stages and the opportunity for radical surgery is restricted. Only if the patients were characterized without other distant metastasis or peritoneal metastasis, they can be treated with surgical resection by the combination of gastrectomy, hysterectomy, and adnexectomy [7]. Most patients can only accept chemotherapy and targeted therapy. However, the clinical characteristics and prognosis of patients with gastric cancer ovarian metastasis (GCOM) remain to be illustrated.

Here, we retrospectively retrieved the GCOM patients' information from the SEER database and analyzed their clinicopathologic characteristics as well as their survival.

## 2. Methods

### 2.1. Data Extraction

Patients in the SEER datasets from 2000 to 2016 were extracted and selected. The including criteria were as follows: (i) ovarian cancer was marked as secondary tumor and (ii) gastric cancer was marked as primary tumor. The exclusion criteria were as follows: (i) the survival time was 0 months and (ii) patients without clarified tumor T stage or N stage.

### 2.2. Data Analysis

Prognosis was evaluated according to both overall survival (OS) and cancer-specific survival (CSS). Survival information was analyzed using the Cox hazard regression model using SPSS Software (version 22.0). Survival nomogram was plotted according to the multivariate survival analysis results.

### 2.3. Nomogram Formulation

Nomograms including clinical features such as age, T, N, and chemotherapy were established to predict GCOM patients' survival possibility at 1-, 3-, and 5-year according to the enrolled cohort.

## 3. Results

### 3.1. Patients' Characteristics

After exclusion, there were 780 cases enrolled in the final cohort (Table 1) with a median follow-up period as 840 days. In brief, 665 (85.3%) cases were white race, 61 (7.8%) cases were black race, and the other 54 (6.9%) cases with other races. Among them, 405 (51.9%) cases were within married status, while the other 375 (48.1%) patients were with single, widowed, or separated status. Only 21 (2.7%) cases were characterized with well-differentiation grade, 77 (9.9%) cases with moderate differentiation, 466 (59.7%) cases with poorly differentiation grade, and 216 (27.7%) cases with undifferentiated grade. Although 296 (37.9%) cases were recorded as an unknown tumor size, 317 (40.6%) cases showed a tumor size larger than 5.0 cm and the other 167 (21.4%) cases with the smaller lesion size. Among them, 55 (7.1%) cases were with the Tx stage, 37 (4.7%) cases with the T0-T1 stage, 63 (8.1%) with the T2 stage, and the other 625 (80.1%) cases with the T3 stage. Half of the patients, 397 (50.9%), were characterized with negative lymph node, 248 (31.8%) with the N1 stage, and 135 (17.3%) with the N2 stage. Up to 664 (85.1%) patients underwent surgical resection, while the other 116 (14.9%) patients did not receive surgical treatment or are unsure. Similarly, 641 (82.2%) cases accepted chemotherapy, and the other 139 (17.8%) refused chemotherapy or are unsure.

### 3.2. Cancer-Specific Survival Analysis

**Figure 1:**
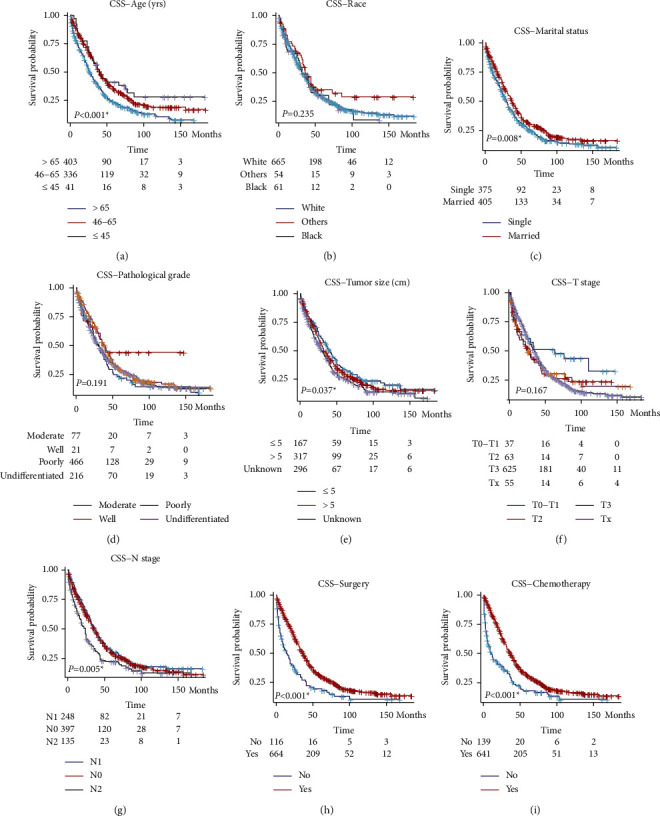
Cancer-specific survival analysis of patients with gastric cancer ovarian metastasis. Survival analyses were conducted using the Cox hazard regression method based on the patients' age (a), race (b), marriage status (c), pathological grade (d), tumor size (e), T stage (f), N stage (g), surgery treatment (h), and chemotherapy (i), respectively.

We next analyzed the cancer-specific survival (CSS) of the enrolled patients (Table 2). Till the end of the follow-up, 568 (72.8%) cases were recorded as cancer-specific death. Univariate analysis revealed the patient's age at diagnosis as a significant prognostic factor. Comparing with patients elder than 65  years old, patients with 46–65  years old and ≤45  years old showed a hazard ratio of 0.683 (95% CI 0.576–0.811, *P* < 0.001) and 0.576 (95% CI 0.388–0.854, *P*=0.006), respectively (Figure 1(a)). As expected, patients' race has no significant effect on the CSS (Figure 1(b), *P*=0.235). Interestingly, the patients within married status showed a better prognosis than those with the single/widowed/separated status (Figure 1(c), HR = 0.799, 95% CI 0.677–0.942, *P*=0.008). The pathological differentiation grade had no statistically significant effect on patients' CSS (Figure 1(d), *P*=0.191). Comparing to patients with the lesion size ≤5 cm, although the patients with the lesion size larger than 5.0 cm showed no significantly different hazard ratio (HR = 1.131, and *P*=0.277), the patients with an unknown tumor size showed a significantly worse CSS (Figure 1(e), HR = 1.327, 95% CI 1.059–1.661, and *P*=0.014). Comparing to those with stage T0-T1, patients with stage T2 (HR = 1.622, 95% CI 0.952–2.764, and *P*=0.075) and T3 (HR = 1.600, 95% CI 1.022–2.506, and *P*=0.040) showed worse CSS, respectively (Figure 1(f)). As expected, patients with the advanced N stage, namely the N2 stage, exhibited significant worse CSS comparing to those with earlier N stages (Figure 1(g), HR = 1.487, 95% CI 1.168–1.894, and *P*=0.001). Survival analyses also revealed that the patients who underwent surgical treatment (Figure 1(h), HR = 0.539, 95% CI 0.429–0.678, and *P* < 0.001) or chemotherapy treatment (Figure 1(i), HR = 0.522, 95% CI 0.422–0.646, and *P* < 0.001) showed smaller cancer-specific death hazard.

**Figure 2:**
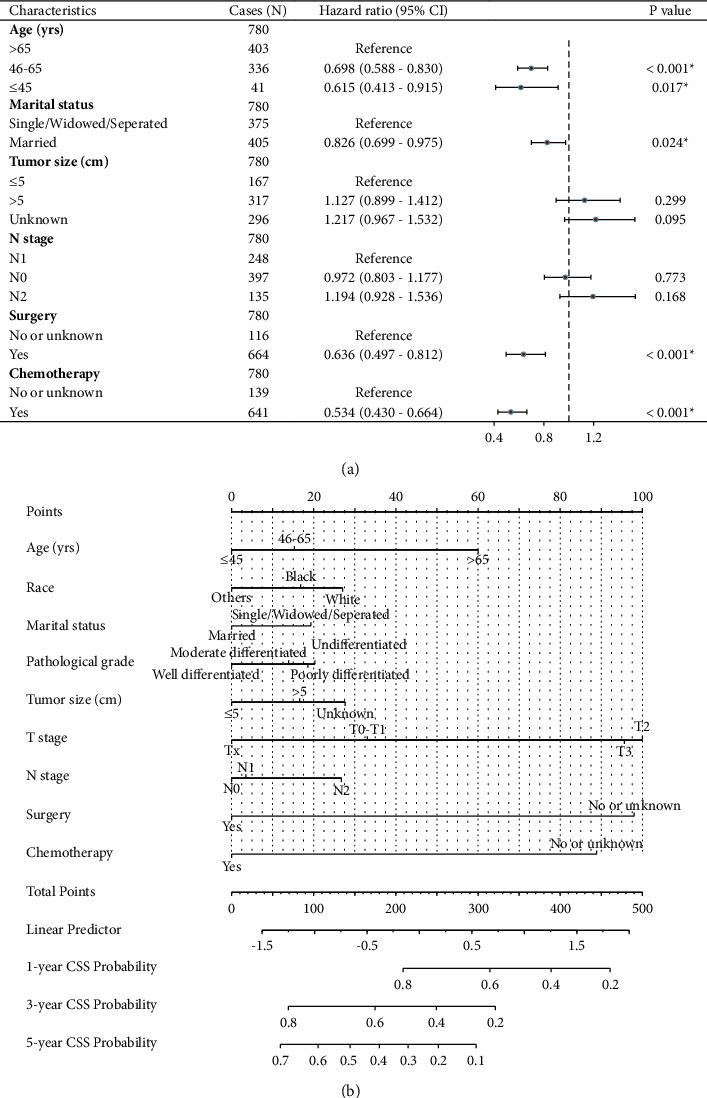
Multivariate analysis and nomogram for cancer-specific survival. (a) Multivariate analysis of cancer-specific survival based on all enrolled variables. (b) Nomogram to predict the 1-, 3-, and 5-year cancer-specific survival possibility of patients with gastric cancer ovarian metastasis.

In addition, we conducted multivariate analysis by subjecting all the significant factors mentioned above (Table 2). Accordingly, younger diagnostic age, married status, undergoing surgical resection, and accepting chemotherapy treatment were identified as four independent prognostic factors. Considering that several variables may have clinical significance although showed no statistical significance in our study, we enrolled all the variables for another multivariate analysis (Figure 2(a)) and established a predicting nomogram for CSS (Figure 2(b)).

### 3.3. Overall Survival Analysis

**Figure 3:**
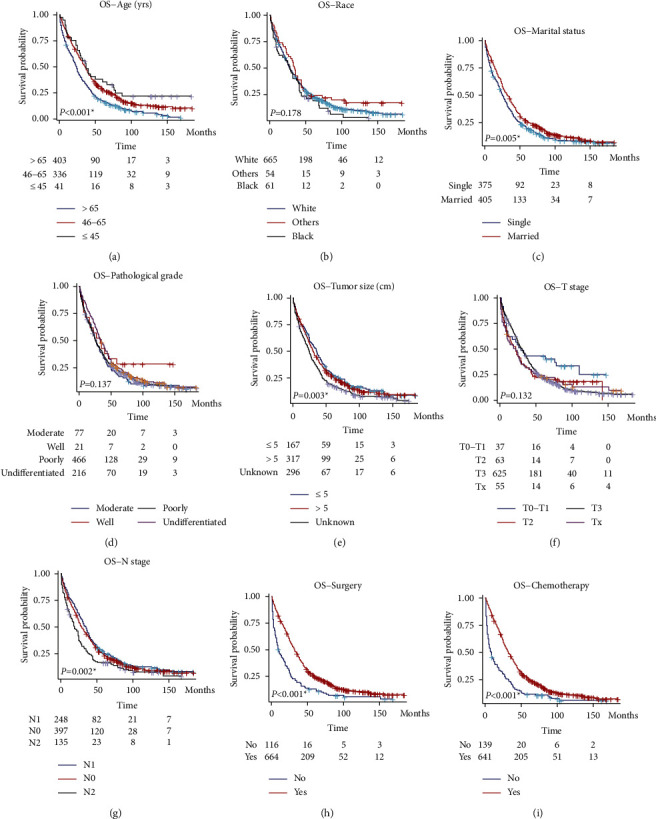
Overall survival analysis of enrolled gastric cancer patients with ovarian metastasis. Survival analyses were conducted using the Cox hazard regression method based on patients' age (a), race (b), marriage status (c), pathological grade (d), tumor size (e), T stage (f), N stage (g), surgery treatment (h), and chemotherapy (i), respectively.

Besides CSS, we also analyzed the overall survival (OS) of the enrolled patients (Table 3). Till the end of the follow-up, 678 (86.9%) cases died. Univariate analysis revealed the patient's age at diagnosis as a significant prognostic factor. Comparing with patients elder than 65 -years-old, patients with 46–65 -years-old and ≤45 -years-old showed a hazard ratio of 0.674 (95% CI 0.576–0.788, *P* < 0.001) and 0.538 (95% CI 0.372–0.777, *P* < 0.001), respectively (Figure 3(a)). As expected, patients' race has no significant effect on the OS (Figure 3(b), *P*=0.178). Interestingly, patients within married status showed a better prognosis than those with the single/widowed/separated status (Figure 3(c), HR = 0.805, 95% CI 0.693–0.937, and *P*=0.005). The pathological differentiation grade had no statistically significant effect on patients' OS (Figure 3(d), *P*=0.137). Comparing to the patients with the tumor size ≤5 cm, although patients with the tumor size larger than 5.0 cm showed no significantly different hazard ratio (HR = 1.077, *P*=0.479), the patients with an unknown tumor size showed a significantly worse OS (Figure 3(e), HR = 1.367, 95% CI 1.115–1.677, and *P*=0.003). Comparing to those with stage T0-T1, patients with stages T2 (HR = 1.609, 95% CI 0.998–2.594, and *P*=0.051), T3 (HR = 1.524, 95% CI 1.020–2.277, and *P*=0.040), and Tx (HR = 1.676, 95% CI 1.031–2.724, and *P*=0.037) all showed worse OS (Figure 3(f)). As expected, patients with the advanced N stage, namely the N2 stage, exhibited significantly worse OS comparing to those with earlier N stages (Figure 3(g), HR = 1.500, 95% CI 1.199–1.875, and *P* < 0.001). Survival analyses also revealed that the patients who underwent surgical treatment (Figure 3(h), HR = 0.514, 95% CI 0.418–0.632, and *P* < 0.001) or chemotherapy treatment (Figure 3(i), HR = 0.506, 95% CI 0.417–0.615, and *P* < 0.001) showed smaller overall death hazard.

**Figure 4:**
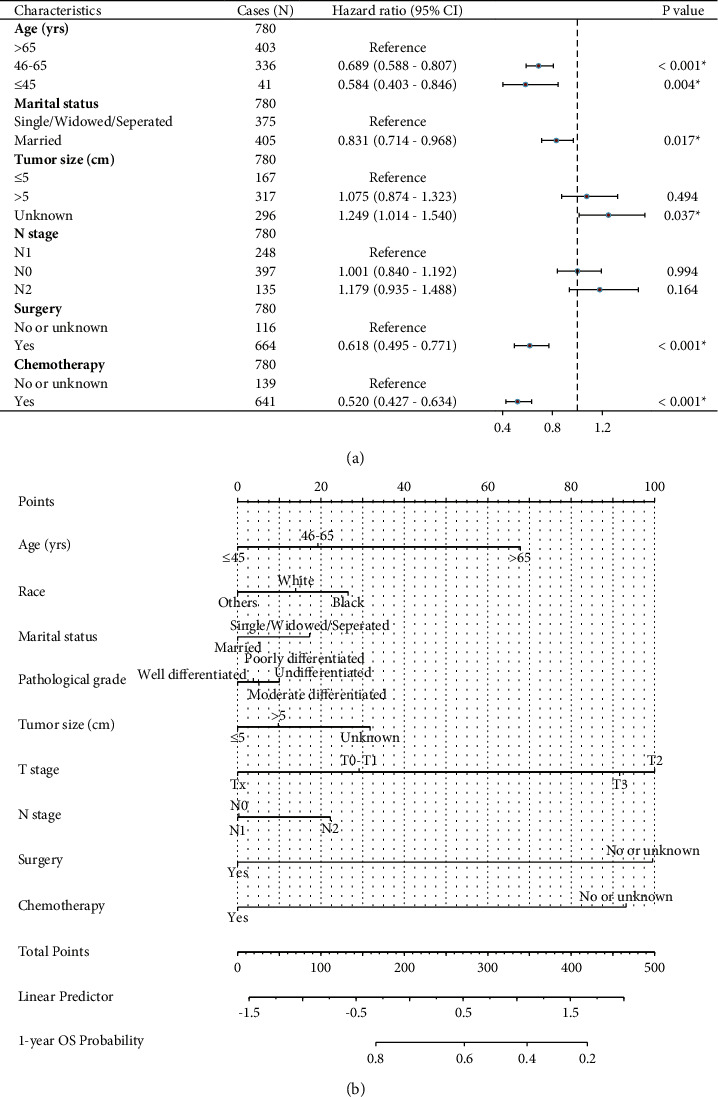
Multivariate cox regression analysis and nomogram for overall survival. (a) Multivariate Cox regression analysis of overall survival based on all enrolled variables. (b) Nomogram to evaluate the cancer-specific survival probability of patients with gastric cancer ovarian metastasis.

In addition, we conducted multivariate analysis by subjecting all the significant factors abovementioned. Consistent with the CSS data, younger diagnostic age, married status, underwent surgical resection, and accepted chemotherapy treatment were identified as four independent overall survival factors. In addition, the patients' tumor size was an independent overall survival predictor (Table 3). Similar with the CSS analyses, we enrolled all the variables for another multivariate analysis (Figure 4(a)) and established a predicting nomogram for OS (Figure 4(b)).

## 4. Discussion

Gastrointestinal cancer metastases to the ovary are a type of ovarian metastases. Ovarian metastatic tumors are most commonly metastasized from gastric cancer to the ovary, accounting for 67% of ovarian metastatic tumors, 5.4% of ovarian malignant tumors, and 1.3% of all ovarian tumors [8, 9]. Among them, Krukenberg tumor caused by the metastasis of signet ring cell carcinoma is an important type of ovarian metastases, and its prognosis is extremely poor [10, 11]. However, few studies reported the clinicopathological characteristics and evaluated the survival of this specific patient group due to the limited sample size.

According to our data, most GCOM patients showed poor pathological differentiation, advanced T stage and N stage. Prognostic variables include the patients' age, tumor size, surgical resection, and chemotherapy treatment. In this specific cohort, several conventional prognostic factors were not significant because the patients were already within the TNM stage IV. This means that several prognostic factors (such as the tumor size) may lose their significant effect on the survival of those patients with very late stage. Liu et al.'s data described the entire younger gastric cancer patients and concluded that early-onset gastric cancer cases showed worse survival compared with late-onset gastric cancers [12], while our data only compared patients with ovarian metastasis. Therefore, the conclusions are completely different. Liu's conclusion was reasonable considering younger patients may have more genetic mutations and more quickly tumor progression. But it is also reasonable in our study that elder patients with distant metastasis exhibited worse prognosis compared than younger advanced-staged patients because elder patients had worse basic health and lower immune capacity to prevent tumor progression. Of note, the marriage status was also identified as an independent prognostic variable for the first time, although the possible underlying mechanisms require further investigation.

In our opinion, surgical resection is suitable for those who are in good general condition, whose primary tumor is resectable or has been resected, and who can tolerate surgery [13, 14]. There are several advantages of surgical treatment. First, diagnosis can be confirmed after operation, so as to prevent patients with primary disease from losing the chance of treatment. Second, surgical resection can reduce compression, inhibit the production of peritoneal effusion, and relieve symptoms. Third, the location and nature of the primary tumor can be clarified and whether it can be resected can be estimated. Lastly, the primary tumor may be resected at the same time and therefore achieve radical treatment and improve patients' survival [15, 16].

Therefore, we suggested that women with gastrointestinal diseases which are considered to be tumors should ask for gynecological consultation or routine pelvic examination. In addition, the pelvis of women patients who are accepting gastrectomy should be routinely explored by surgeons. Besides, women with a history of gastrointestinal tumor surgery should have a regular gynecological follow-up. For patients with limited pelvic metastases, total hysterectomy and bilateral adnexectomy can be performed to remove pelvic metastases as much as possible [17].

Our study has several limitations. First, the SEER dataset includes data from a limited number of geographic regions and may not be the representative of the overall US population. This could result in an overrepresentation or underrepresentation of certain races, which could affect the accuracy of survival analyses based on race. Second, while the SEER dataset contains a large amount of data, some subgroups of interest may have relatively small sample sizes such as our specific cohort, and the limited sample size request us to validate the major conclusion in more cohorts worldwide in the future. Consistently, to make our data more precise, we selected a strict data exclusion strategy and may thus missed information on important variables. Finally, here we did not analyze the survival effect of comorbidities, which cannot be obtained from the SEER dataset. However, comorbidities affect patients' survival and treatment outcomes. Without this information, it can be difficult to account for the impact of comorbidities on survival and accurately assess the effectiveness of different treatments.

## 5. Conclusion

Taken together, our data suggested that the diagnostic age, marriage status, surgical resection, and chemotherapy treatment were significant prognostic factors for gastric cancer patients with ovarian metastasis.

## Figures and Tables

**Table 1:** Patients' characteristics.

Characteristics	Cases
*Race, n (%)*	
White	665 (85.3%)
Others	54 (6.9%)
Black	61 (7.8%)

*Marital status, n (%)*	
Single/widowed/seperated	375 (48.1%)
Married	405 (51.9%)

*Pathological grade, n (%)*	
Moderately differentiated	77 (9.9%)
Well differentiated	21 (2.7%)
Poorly differentiated	466 (59.7%)
Undifferentiated	216 (27.7%)

*Tumor size (cm), n (%)*	
≤5	167 (21.4%)
>5	317 (40.6%)
Unknown	296 (37.9%)

*T stage, n (%)*	
T0-T1	37 (4.7%)
T2	63 (8.1%)
T3	625 (80.1%)
Tx	55 (7.1%)

*N stage, n (%)*	
N1	248 (31.8%)
N0	397 (50.9%)
N2	135 (17.3%)

*Surgery, n (%)*	
No or unknown	116 (14.9%)
Yes	664 (85.1%)

*Chemotherapy, n (%)*	
No or unknown	139 (17.8%)
Yes	641 (82.2%)

*CSS, n (%)*	
1	568 (72.8%)
0	212 (27.2%)

*OS, n (%)*	
1	678 (86.9%)
0	102 (13.1%)
Days, median (IQR)	840 (330, 1597.5)

**Table 2:** Cancer-specific survival of gastric cancer patients with ovarian metastasis.

Characteristics	Cases (*N*)	Univariate analysis		Multivariate analysis	
		Hazard ratio (95% CI)	*P* value	Hazard ratio (95% CI)	*P* value
Age (yrs)	780		<0.001^*∗*^		
>65	403	Reference		Reference	
46–65	336	0.683 (0.576–0.811)	<0.001^*∗*^	0.698 (0.588–0.830)	<0.001^*∗*^
≤45	41	0.576 (0.388–0.854)	0.006^*∗*^	0.615 (0.413–0.915)	0.017^*∗*^
Race	780		0.235		
White	665	Reference			
Others	54	0.752 (0.531–1.064)	0.108		
Black	61	1.028 (0.744–1.419)	0.868		
Marital status	780		0.008^*∗*^		
Single/widowed/seperated	375	Reference		Reference	
Married	405	0.799 (0.677–0.942)	0.008^*∗*^	0.826 (0.699–0.975)	0.024^*∗*^
Pathological grade	780		0.191		
Moderately differentiated	77	Reference			
Well differentiated	21	0.583 (0.307–1.109)	0.100		
Poorly differentiated	466	0.924 (0.702–1.217)	0.574		
Undifferentiated	216	0.816 (0.607–1.099)	0.181		
Tumor size (cm)	780		0.037^*∗*^		
≤5	167	Reference		Reference	
>5	317	1.131 (0.906–1.413)	0.277	1.127 (0.899–1.412)	0.299
Unknown	296	1.327 (1.059–1.661)	0.014^*∗*^	1.217 (0.967–1.532)	0.095
T stage	780		0.167		
T0-T1	37	Reference			
T2	63	1.622 (0.952–2.764)	0.075		
T3	625	1.600 (1.022–2.506)	0.040^*∗*^		
Tx	55	1.662 (0.964–2.864)	0.068		
N stage	780		0.005^*∗*^		
N1	248	Reference		Reference	
N0	397	1.047 (0.869–1.261)	0.628	0.972 (0.803–1.177)	0.773
N2	135	1.487 (1.168–1.894)	0.001^*∗*^	1.194 (0.928–1.536)	0.168
Surgery	780		<0.001^*∗*^		
No or unknown	116	Reference		Reference	
Yes	664	0.539 (0.429–0.678)	<0.001^*∗*^	0.636 (0.497–0.812)	<0.001^*∗*^
Chemotherapy	780		<0.001^*∗*^		
No or unknown	139	Reference		Reference	
Yes	641	0.522 (0.422–0.646)	<0.001^*∗*^	0.534 (0.430–0.664)	<0.001^*∗*^

**Table 3:** Overall survival of gastric cancer patients with ovarian metastasis.

Characteristics	Cases (*N*)	Univariate analysis		Multivariate analysis	
		Hazard ratio (95% CI)	*P* value	Hazard ratio (95% CI)	*P* value
Age (yrs)	780		<0.001^*∗*^		
>65	403	Reference		Reference	
46–65	336	0.674 (0.576–0.788)	<0.001^*∗*^	0.689 (0.588–0.807)	<0.001^*∗*^
≤45	41	0.538 (0.372–0.777)	<0.001^*∗*^	0.584 (0.403–0.846)	0.004^*∗*^
Race	780		0.178		
White	665	Reference			
Others	54	0.819 (0.603–1.113)	0.203		
Black	61	1.194 (0.903–1.579)	0.213		
Marital status	780		0.005^*∗*^		
Single/widowed/seperated	375	Reference		Reference	
Married	405	0.805 (0.693–0.937)	0.005^*∗*^	0.831 (0.714–0.968)	0.017^*∗*^
Pathological grade	780		0.137		
Moderately differentiated	77	Reference			
Well differentiated	21	0.669 (0.383–1.169)	0.158		
Poorly differentiated	466	0.946 (0.735–1.218)	0.668		
Undifferentiated	216	0.806 (0.613–1.060)	0.124		
Tumor size (cm)	780		0.003^*∗*^		
≤5	167	Reference		Reference	
>5	317	1.077 (0.878–1.320)	0.479	1.075 (0.874–1.323)	0.494
Unknown	296	1.367 (1.115–1.677)	0.003^*∗*^	1.249 (1.014–1.540)	0.037^*∗*^
T stage	780		0.132		
T0-T1	37	Reference			
T2	63	1.609 (0.998–2.594)	0.051		
T3	625	1.524 (1.020–2.277)	0.040^*∗*^		
Tx	55	1.676 (1.031–2.724)	0.037^*∗*^		
N stage	780		0.002^*∗*^		
N1	248	Reference		Reference	
N0	397	1.092 (0.921–1.295)	0.312	1.001 (0.840–1.192)	0.994
N2	135	1.500 (1.199–1.875)	<0.001^*∗*^	1.179 (0.935–1.488)	0.164
Surgery	780		<0.001^*∗*^		
No or unknown	116	Reference		Reference	
Yes	664	0.514 (0.418–0.632)	<0.001^*∗*^	0.618 (0.495–0.771)	<0.001^*∗*^
Chemotherapy	780		<0.001^*∗*^		
No or unknown	139	Reference		Reference	
Yes	641	0.506 (0.417–0.615)	<0.001^*∗*^	0.520 (0.427–0.634)	<0.001^*∗*^

## Data Availability

Data used to support the findings of this study are available from the corresponding author upon request.
